# Editorial

**Published:** 1863

**Authors:** 


Amaurosis and Smoking.—At the Royal London Ophthalmic Hospital, a severe case of amaurosis was lately presented, evidently brought on by the immoderate smoking of tobacco. Examined with the ophthalmoscope, both optic nerve disks were found partially atrophied; the apparent inner half of each white, the outer red and hyperæmic. Mr. Wordsworth pointed out the case to the class as one of "tobacco amaurosis," of which he had lately seen several in excessive smokers, with similar symptoms. This form of amaurosis he considered quite incurable.—*Boston Med. & Surg. Journal.*

---

Calomel and Tartar Emetic.—At a meeting of the regular medical profession of Cincinnati, called to consider the late Circular No. 6 of the Surgeon-General of the United States, in reference to the use of calomel and tartar emetic in the Army, a committee was appointed to report the sense of the profession on the subject. At a subsequent meeting, the committee reported, denying the truth of the statements on which the Surgeon-General's order was founded, charging him with the assumption of powers not belonging to his office, and ending with a resolution that his removal therefrom would meet the approbation of the profession, be of advantage to our soldiers, and creditable to the Government.—*Ibid.*

### Editorial.

### AMERICAN MEDICAL ASSOCIATION.

Notwithstanding the timidity of some, the open opposition and misrepresentations of others, and the disturbing influences of civil war, the recent meeting of the Association in this city was eminently successful. More than two hundred members were present, representing seventeen States and Territories; and among them some of the most eminent of the profession. As will be seen by the Record of Proceedings, contained in the present number of the *Examiner*, the attention of members was wholly absorbed with the proper business of the Association. Not a word or an act of a sectional or political character disturbed its deliberations; and no one deemed the *patriotism* of its members sufficiently *questionable* to require the adoption of special resolutions to bolster it up, or herald it to the world.

The great lack of reports from Special Committees was very well supplied by the voluntary communications of Drs. J. H. Griscom, H. G. Davis, and J. Homberger, of New York; and Drs. E. Andrews and A. Fisher, of Chicago. The two last-mentioned papers led to some interesting and profitable discussion, in which several members of the Association participated. There was a well-written and interesting Report from the Standing Committee, on Medical Education, by Dr. C. C. Cox, of Maryland. It was listened to with attention; and the resolutions appended to it, after a slight amendment, were adopted. The topic which elicited most discussion was the Surgeon-General's order, excluding calomel and tartarized antimony from the supply-table for the Medical Department of the Army. This topic, after receiving the careful consideration of a large committee, elicited an animated, though courteous and candid, discussion. The resolutions condemning the order and asking for its recision or withdrawal were passed, with but few dissenting voices. Our views on this subject are given in another page of this *Journal*. But no part of the doings of the recent meeting of the Association afforded us, personally, as much satisfaction as the selection of officers for the ensuing year. It is well known that the Association, at an early period of its history, undesignedly adopted the *custom* of selecting the president from the place where the annual meeting was held. This custom, once established, as completely restricted the selection of candidates for the highest honors of the Association to the profession of a few large cities, as though all the rest were excluded by positive constitutional provisions. A custom so contrary to the national character of the organization, and so unjust to the greater part of the profession, met with early and continuous opposition from some of the warmest friends of the Association; and from none more decidedly than the editor of this *Journal*. From motives of delicacy, however, each successive nominating committee (with one exception,) followed the custom,—simply nominating whoever was recommended by the representatives of the local profession in the city where they were holding the meeting. Aside from the partiality and injustice of such a custom, it induced other evils of scarcely less magnitude. Thus, no sooner was a place selected for the next Annual Meeting, than every prominent member of the profession in that locality became suspected of being a candidate for the next presidency; and if the local profession did not actually split into factions,—each striving to bring its own favorite into a position for nomination, they were subject to the annoyance of being constantly so represented by half of the medical periodicals in the country. Impressed with the importance of breaking up such a custom, the following resolutions were presented to the meeting of the Illinois State Medical Society in the spring of 1859. They were laid on the table for further consideration. At the next annual meeting, in May, 1860, they were taken from the table, fully considered, and adopted by the Society with only two dissenting voices. Notwithstanding this action of the State Medical Society, in which representatives of all the Medical Societies and Colleges of this city participated, no sooner was this city selected as the place for an annual meeting, than the profession here was represented as divided into factions, under the lead of two rival candidates for the presidency; and, if a medical journal, published in Louisville in the winter of 1860 and '61 was to be credited, these two parties were waging a fiercer war upon each other than that now carried on in *Rebeldom.*

It was in vain that we denied the existence of any such factions or candidates. Their existence continued to be alleged, and believed in, up to the hour of the recent meeting of the Association in this city. Indeed, so serious were these local feuds supposed to be, that some of the oldest members of the Association in Philadelphia, New York, and other distant cities, actually entertained fears for the welfare of the organization; and some professional *Ishmaelite*, who never had a truly *patriotic* idea in his head or impulse in his heart, even took the trouble to set afloat in the medical journals the wonderful idea, that the *loyalty* of some supposed candidate was not like the virtue of Cæsar's wife, entirely "above *suspicion.*" We can well imagine the surprise of the members of the Association, when, on their arrival in this city, they found one of the supposed candidates for the presidency so much engaged in projects for enlarging *canals* that he neither deigned to look at the Association nor to open the doors of his house for the reception of one of its members; while the greatest anxiety of the other was to get the following letter and resolutions fairly before the Nominating Committee in time, effectually to break up the only absurd custom which had fastened itself upon the annual doings of our national organization:—

CHICAGO, *June* 1*st*, 1863.

"To the Delegate on the Nominating Committee from Illinois:

"Dear Sir:—Enclosed you will find a series of Resolutions in relation to the custom of selecting a President for the American Medical Association from the place where the meeting is held, adopted by an almost unanimous vote of the Illinois State Medical Society, at the annual meeting in May, 1860.

"These Resolutions still stand as instructions to the Delegates from that Society. Should my name be mentioned in the

Nominating Committee as a candidate for President of the Association, you will confer a great favor on me by immediately presenting these Resolutions and this note, with the assurance that under no circumstances, can I permit myself to accept a nomination for the Presidency of the American Medical Association while the *custom* alluded to in the Resolutions is in force." Yours truly.

N. S. DAVIS,

*Permanent Sec'y of the Ill. State Med. Society.*

"*Whereas*, The American Medical Association is a national Association, composed of delegates and members from all parts of the United States, meeting on terms of perfect equality:—

"Therefore, *Resolved*, That, in the opinion of this Society, all the officers of the Association should be selected strictly with reference to *merit*, and without any regard to their place of residence.

"*Resolved*, That the custom of selecting the President of the Association exclusively from the profession of the city in which the Annual Meeting is held, is not only derogatory to the general character of the organization, and calculated greatly to lessen the honor which should attach to that office, but past experience has shown that it leads directly to local divisions, jealousies, and injurious partisan strife.

"*Resolved*, That the delegates from this Society to the Association, be instructed to use their influence to abrogate the custom alluded to in the preceding resolution.

"*Resolved*, That the Secretary be directed to furnish copies of the foregoing resolutions to other State and Local Medical Societies, and ask their attention to the same."

The worthy representative on the Nominating Committee from Illinois performed his duty well, by presenting the documents at the right time, and thereby relieved the Committee from all feelings of delicacy, and compelled it to go to the profession at large for a candidate for its highest honor.

We thus had the extreme gratification of seeing the great American Medical Association again in active *harmonious* operation; of taking its members by the hand at our own fireside; and of striking the death-blow to a custom which we trust will never be revived.

Calomel and Tartarized Antimony in the Army.—No subject has recently elicited more discussion in the profession than the Order of the Surgeon-General, excluding calomel and tartar emetic from the supply-table of the Army. If, in a general revision of the list of medicines to be furnished on the requisition of army-surgeons, the Surgeon-General had simply omitted calomel, and tartrate of antimony and potassa, it would, probably, have attracted very little attention from the profession, generally. But the issuance of a special order, excluding these articles on the pretext that the first, especially, had been so grossly and generally abused by the medical officers of the Army, as to admit of no other mode of correction; and that recent improvements in pathology had rendered both unnecessary if not always hurtful, was, to say the least, not very modest in the Surgeon-General,—nor very complimentary to the medical staff over which he presides.

And, when we remember that a very large part of the present medical staff of the Army has been taken recently from the great mass of active practitioners throughout the country, it is not surprising that such an Order should excite general criticism. The attempt to correct the abuse of a remedial agent of acknowledged active qualities, by prohibiting its use, is so unphilosophical and absurd, that we confess to a feeling of chagrin at the thought, that one in the high and honorable position of Surgeon-General of the United States Army, should place himself on record in that position before the medical world. That he should go further, and, because some of the medical officers were represented to have abused these articles, therefore, publish to the world, substantially, that the whole medical staff of the United States Army were incapable of being safely trusted with the use of two of the oldest and most common articles of the *Materia Medica*, would be humiliating indeed, if it were not so manifestly rediculous. The pretence set up by those who attempt to defend the Surgeon-General, that, in excluding calomel, he did not intend to exclude all mercurials, but left still in the supply-table blue mass, hyrag. cum. creta, the corrosive chloride, and the iodides, only make the matter worse.

For certainly no intelligent practitioner will pretend that corrosive sublimate, the iodides of mercury, or even blue mass, are either more mild or uniform in their action on the human system, than calomel; or that medical officers can be safely trusted with the former, who are too ignorant or careless to manage the latter with safety to their patients. Without any reference to the merits or demerits of calomel and antimony as remedial agents in the treatment of disease, the reasons set forth in the order for this prohibition are so manifestly unjust and unphilosophical, that we can see but two ways by which the Surgeon-General can escape the well-merited contempt of the profession. One is by acknowledging that the Order was issued without due consideration, and promptly withdrawing it; and the other is by immediately publishing the actual statistics of *salivation* and *mercurial gangrene* in the army, specifying the regiments and hospitals in which the cases occurred, and also the statistics claimed to be in his office, demonstrating that tartar emetic, as well as calomel, can be advantageously dispensed with in the treatment of all diseases of the army.

If the Surgeon-General refuses to adopt the first course of action, the profession certainly have a right to demand the adoption of the latter.

---

Hospitality of the Profession in Chicago.—A correspondent of the *American Medical Times* writes concerning the hospitalities extended to members of the American Medical Association, during its recent session in this city, as follows:—

"I cannot close this letter without bearing testimony to the liberal and generous hospitality of our professional brethren of Chicago. Delightful evening entertainments were given by Drs. N. S. Davis, W. W. Allport, W. H. Byford, M. Parker, A. Groesbeck, W. Carr and Son, and J. P. Ross. These entertainments were well attended, not only by the delegation, but also by Chicago feminine beauties. Your correspondent is deeply indebted to the profession of Chicago, and their families, for many a happy hour. Yours,

Correspondent."

CHANCRES.—*By W. E. Bowman, M.D.*—Treatment of Soft Chancre.—Assuming that the reader is cognizant of the facts so briefly stated in the last two numbers of the *Lancet*, I need not dwell on the importance of a proper diagnosis of the different forms of chancre, before commenting on the treatment of them, which differs so widely.

Although mercury, taken internally, ends the cicatrization of hard chancre, it has no beneficial influence upon the chanceroid, which remains stationary or even progresses after salivation.

The virus, resting in the sore itself and its underlying tissues, is only effectually destroyed by thorough cauterization.

*Pernitrate of mercury.*—Having been, invariably, successful with this form of caustic for the arrest of soft chancres, in my own practice, I place it "par excellence," first on the list. I prepare it by adding an ounce of red precipitate to an ounce and a-quarter of nitric acid, in which it readily dissolves by shaking. It is very painful when thoroughly applied, causing much inflammation; and, when the chancre is large, the effusion of serum into the cellular tissue of the prepuce. It has seldom to be employed but once, however, even in aggravated cases; nor have I ever noticed any injurious effect, hitherto, from its employment. Linseed poultices should be kept to the part until the inflammation subsides, and, afterwards, water-dressing; when the gray slough separates, which it does, generally, in three or four days, the healthy ulcer left, afterwards, must be treated in the usual way with wet lint and oiled silk; stimulating it with red wash or solution of the chlorate of potash, should the granulations become exhuberant. Collections of serum, formed after the operation, may be allowed to ooze away through punctures made into them with a needle.

*Canquoin's paste.*—Rollet and Diday assert that this caustic, composed of equal parts of chloride of zinc and flour, whilst exceedingly efficacious, gives but very little pain. It is made by drying the powdered chloride over a spirit-lamp before mixing it with dried flour, and adding alcohol drop by drop until the paste is formed, which is to be spread thinly on a cloth and again subjected to a gentle heat, a disc of this paste, corresponding in shape to the chancre and slightly exceeding in size, is cut out and retained upon the surface, previously cleansed of matter, from one to three hours, and, in large phagedenic ulcers, from four to six hours, the patient keeping his bed until the paste is removed.

*Other caustics.*—Nitric, strong acetic, and sulphuric acids, caustic soda, potassa cum calce, and even the actual cautery or knife have their respective advocates. Dr. Bumstead, to whose work much of our former article was indebted, recommends the nitric acid in preference to all other applications, although he confesses that it sometimes requires to be repeated every second or third day.

*When wrong to cauterize.*—Thorough cauterization is inadmissible when chancroid extends deeply, and is situated directly over the urethra in either male or female, or in the vagina, when lying in contact with the bladder, rectum, or peritoneum, on account of the danger of an opening being created into these parts on the separation of the slough.—Again, cauterization is not applicable when the chancroid cannot be fully exposed as in phymosis, or when situated within the urethra, os uteri, &c., and would be useless unless every ulcer could be reached that would be likely to inoculate anew the eschar.

*Nitrate of silver.*—This is altogether too feeble in its action for universal adoption in cases of chancroid, but proves extremely useful in those enumerated that do not allow of a more powerful application. A comparative trial of the merits of the nitrate of silver and the solution of the pernitrate of mercury, would satisfy the most sceptical of the superiority of the latter, for the sore which has long remained stationary or even continued to extend, notwithstanding the constant use of the one, will be found to yield rapidly and cicatrize after a single thorough employment of the other.

*Stimulating lotions.*—These have the same influence upon a chancre as upon simple ulcers, and although they do not affect its specific character, do much good by keeping the pus removed as fast as it is secreted, and by coagulating the virus and hardening the adjacent tissues, prevent the inoculation of the surrounding parts and check the growth of the sore.

Among the many astringent and disinfecting lotions now in vogue, the following may be mentioned as some of those most frequently employed, viz.:—

℞. Zinci chlor. gr. j. aquæ ℥j. m.

℞. Liq. sodæ chlorinatæ ʒj. aquæ ℥ij. m.

℞. Ac. nitrici dil. ʒj. aquæ ℥viij. m.

℞. Tannin ʒij. tinct. opii. ℥ss. aquæ ℥viij. m.

But the strength of these solutions must be adapted to the sensibility of the part, which varies in different cases, they should never be so strong as to excite pain or produce irritation, and indeed in many cases, when constant attention can be paid to them, the lotion might as well consist entirely of water or glycerine.

The dressings should be kept covered with oiled silk, and renewed, in ordinary cases, as often as two or three times a day, that the discharges should not long remain in contact with the sore.

The black-wash, so much employed all over the world, is composed of two scruples of calomel and four ounces of lime-water. It is less cleanly and desirable than any of the forms above-mentioned.

Acetate of lead is objectionable, on account of its forming an insoluable albumenate of lead on the surface of the sore, which is with difficulty removed, and hides its progress.

Chancres beneath the prepuce, when it can be drawn back and examined, are often dressed with dry lint, which soon becomes sufficiently moistened by the natural secretion of the part.

*Chancres of the frœnum.*—The frænum is particulary liable to be destroyed by chancre. When perforation takes place, the bridle should be cut and the raw surfaces cauterized. Diday recommends the separation to be made with a pair of scissors, which should be dull, these cut and cauterize at the same moment.

*Urethral chancres.*—The surfaces of urethral chancres, when near the meatus, should be kept separate by means of wet lint, which should be pushed down upon the sore with a probe, and have a thread attached to it to facilitate its withdrawal. When out of sight, the case must be treated as in gonorrhœa, by first subduing the inflammatory symptoms, by diet, rest, diluents, cathartic medicines, &c., and the employment of emollient urethral injections, afterwards resorting to those which are more powerful.

*Phymosis.*—If the chancroid be concealed by a tight and inflamed prepuce, free use should be made of the syringe, with tepid bathing of the part, which will not only keep the secretion from collecting, but also contribute materially to the reduction of the inflammation. When possible, a little dry lint may be passed up to the sore and allowed to remain for a few hours before removal. When the head of the penis is swollen and painful, it must be kept constantly buried in an emollient poultice or be fomented with infusion of poppy heads.

*Iron, internally.*—When soft chancres are slow in healing, Dr. Thompson remarks, that nothing appears to hasten cicatrization so much as a mild form of iron given internally, and the potassio-tartrate appears to him to be the most successful in such cases; he prescribes it in doses of a scruple, in water, twice a day.—*Canada Lancet.*

---

ON THE TREATMENT OF CONSUMPTION BY THE HYPOPHOSPHITES.

To the Editor of *The Lancet:*

Sir:—Dr. Cotton's recent experiments are only a repetition of his former ones, with the time extended from a fortnight to a month. They are as invalid as the first.

He begins by asserting that the hypophosphites have no physiological action. If he will take an overdose, say four or five tablespoonfuls daily, of the syrup, he will soon find, to his very great discomfort, they produce such a state of plethora as to bring on epistaxis, or hæmoptysis, or bleeding from the intestines, either with or without symptoms of congestion towards the head or lungs. His principle is to give the hypophosphites for a month, then to leave them off to give some other substance, claiming for this second substance the merit of whatever improvement may continue after the suspension of the hypophosphites. This rests upon the assumption that the effect of the hypophosphites ceases as soon as the medicine is suspended, which is contrary to experience with regard to the action of all mineral substances assimilated by the organism. Upon the same principle, Dr. Cotton would have the right to conclude that if a patient who has been vaccinated begins by taking carbonate of soda immediately after, and continues it for some time, his immunity from small-pox would be owing to carbonate of soda, and not to vaccination.

The most elementary principle of experiment is to distinguish and isolate, as far as possible, the different conditions upon which the result of the experiment depends, so as to ascertain those which really contribute to the result, and those which do not. Dr. Cotton is, evidently, acquainted with this principle; for not only does he not apply it, but willfully and pre-determinately violates it in every one of his experiments. If his object had really been to ascertain the true value of the hypophosphites in the treatment of consumption, the simple, rational, logical, and scientific plan would have been to take a series of patients in certain determinate pathological conditions, and to treat them with the hypophosphites until death or cure ensued. During the same period another series of patients, selected as nearly as possible in the same pathological condition as the first, should be submitted to some other course of treatment. These two series of researches, if scientifically carried out, would have afforded more data for comparison, and allowed of some definite conclusion. But, as Dr. Cotton's object was rather to play the part of advocate, and to find a plea against the efficacy of the hypophosphites, he has taken care to produce no well-defined and scientific results. He has, nevertheless, defeated the purpose he had in view, and produced a certificate in favor of the hypophosphites; for, according to his own showing, out of 12 cases (of which 2 are declared beforehand to be unpromising, thus reducing the number to 10), the hypophosphites were successful in 7 instances, producing improvement, and much improvement, and moderate improvement; the weight of the patients increasing in every instance, and in one or two to a great extent.

For the future, I would ask Dr. Cotton, before he institutes any further experiments in therapeutics, to bear in mind the following question:—How many negative results in experimental science, obtained under variable or unknown conditions, are sufficient to overthrow one single positive instance, the character and condition of which have been scientifically determined? When he has thought of this question in all its bearings, he will have a better right to come forward as a therapeutist; for such a mode of proceeding in therapeutics, as that followed by Dr. Cotton, appears to me to have no more claim to be a scientific experiment, than would be the fact of a man, ignorant of chemistry, going into a laboratory and throwing half-a-dozen of the first substances he met with into a crucible, and calling that a chemical experiment. The hypophosphites have proved themselves to any impartial observer, even, as I conceive, in the hands of Dr. Cotton himself, to be medicines of the highest importance. Men of scientific attainments and practical experience prescribe them, and will, I have no doubt, continue to prescribe them, to the relief of patients suffering from one of the most intractable of maladies which afflict humanity.

I remain, Sir, your obedient servant,

J. F. Churchill.

Avenue, Montaigne, 1863.

—*London Lancet.*

---

Iodide of Lime.—A Substitute for Iodide of Potassium.—*By James R. Nichols, Chemist.*—The "Iodide of Lime," first introduced in 1855 by Dr. Puddock, a distinguished London physician, has been rapidly gaining favor among English practitioners, as a remedy of great value. It is used in those cases where iodide of potassium is indicated with more marked effects than usually attend the use of that salt. The success attending its use has led us to prepare the article with much care; and also to present it in aqueous solution,—which is, perhaps, a preferable form. The lime and iodine are held together by a very feeble affinity, and the salt will not admit of exposure without evolving free iodine. The *solution* is a colorless and almost tasteless liquid, and remains permanent although long kept and exposed to the air.

Each drachm of the salt contains eight and a-half grains of iodine; and each fluid ounce of the solution contains half a grain of iodine. The iodine, *in the solution*, exists in the form of Iodide of Calcium and Iodate of Lime, thus:—$6CaO=6I+5CaI+CaO, IO^5$. Acids decompose the solution, and free the iodine; hence the utility of this form for the administration of iodine, probably, in the state of an oxide. The Iodide of Lime is superior to Iodide of Potassium in several particulars, as,

1.—The smallness of the dose, and the minute state of its atomic division. 2.—In not passing off so quickly through the kidneys. 3.—In its ready combination with the blood and tissues, manifested by its alterative effects. 4.—In being nearly tasteless, and, therefore, readily taken by children. 5.—In being much less expensive. 6.—In not producing either gastro-enteritic or vesical irritation.

Iodide of Potassium is an expensive remedy; and, on that account, many physicians refrain from using it. The Iodide of Lime being found to be more efficacious and much cheaper, will, doubtless, be substituted for it to a great extent. It has been used in England, with much success in throat diseases, in morbid conditions of the general system, in scrofulous affections, in intractable cases of neuralgia, in diseases caused by metallic poisons, etc. At the Bloomsbury Dispensary, England, it has been extensively prescribed for three years, with much success.

The dose of the salt is very small,—about one-fourth of a grain two or three times a day. Of the solution, two to four fluid drachms may be given as often. The salt is put up in ounce bottles, and the solution in pounds. The iodine in the solution is set free by adding a few drops of nitric, sulphuric, or hydrochloric acid. Druggists and physicians will please institute the experiment, that the presence of Iodine may be demonstrated in the clear solution. Physicians ordering our article, may rely upon its purity and accuracy of combination. Physicians should order the salt in one-ounce vials, and prepare the solution themselves. Directions accompany each vial.

Materia Medica and Hygiene in the University of Buffalo.—We understand that Prof. Charles A. Lee, having completed his European tour and returned to this country, resumes the duties of his Professorship in the University of Buffalo, and will lecture at the approaching term upon *Materia Medica* and Hygiene.—*Buffalo Journal.*

---

Diphtheria.—Dr. C. V. Moore, of Stillwater, N. J., writes:—"Diphtheria has been prevailing again, the type sthenic. It yields, pretty generally, under the administration of the sulphate of zinc, in emetic doses repeated every twenty-four or thirty-six hours, in conjunction with chlorate of potash and tincture of the chloride of iron. Pustulations, with croton oil to the neck, seemed decidedly beneficial, I have met with several cases that recovered unexpectedly, even after the extension of the disease to the larynx, accompanied by croupy cough." Dr. Moore speaks highly of the beneficial effects of the zinc emetics in these cases.—*Medical and Surgical Reporter.*

---

The Action of Expectoration.—In a paper on this subject read at a recent meeting of the Glasgow Medical and Chirurgical Society, by Dr. W. T. Gairdner, he advanced the theory, that the bronchial tubes acted in a manner similar to that of the bowels, and ejected their contents by a peristaltic motion, which could as certainly be increased by appropriate medicines as could that of the bowels.—*London Lancet.*

---

Academy of Medicine, Paris.—Professor Rokttansky has been elected a Foreign Associate by forty-two out of fifty-three voters present. The other candidates were Virchow, Frerichs, and Magnus Huss.—*London Lancet.*

---

Vivisection.—The deputation of the Society for the Prevention of Cruelty to Animals, which some time since waited on the Emperor Napoleon to protest against the practice of vivisection, have been rewarded by an ordinance of police, which places a check on this practice. The veterinary and anatomical schools are, therefore, expected to relinquish it entirely.—*London Lancet.*

---

Cinchona in Java.—The Dutch Government, having prohibited the culture of opium, has wisely favored the production of the cinchona, imposing upon the planters such regulations as are necessary to the preservation of the trees.—*Ibid.*

RANK OF NAVAL SURGEONS.—By a recent order, issued from the Navy Department, the following is to be the rank of surgeons in the navy:—

Surgeon of the Fleet to rank with Captain.

Surgeons to rank with Lieutenant-Commanders, for the first five years after promotion; after the first five years with Commanders; and after fifteen years to rank with Captains.

Passed Assistant-Surgeons to rank with Lieutenants.

Assistant-Surgeons to rank with Masters.—*Medical and Surgical Reporter.*

---

DIMINISHED DEATH-RATE IN THE ARMY.—From 1830 to 1836 the annual death-rate among the troops in the United Kingdom was 14 per 1000; in the years 1859-60 it was reduced to 5. During the same periods, the death-rate in the cavalry of the line was reduced from 15 to 6; in the royal artillery, from 15 to 7; in the foot guards, from 21 to 9; in the infantry of the line, from 17 to 8. A similar decrease is to be reported respecting the British troops in the Colonies.—*London Lancet.*

---

THE DEATHS IN LONDON IN 1862 from all causes were 66, 950, of which 34,133 were males, and 32,817 females. The greatest number occurred in the forty-eighth week, ending 29th of November, when they rose to 1,745. In that week the mean temperature of the air fell to 37·1°. The least number occurred in the twenty-eighth week, and was 1,065, when the mean temperature rose to 58·2°.—*London Lancet.*

---

PERUVIAN PHYSICIANS.—Dr. Markham, in his travels in Peru, describes the physicians as a wandering class. With their wallets of drugs on their backs and dressed in black britches, a red ponchof and broad-brimmed hat, they walk in direct line from village to village, as did their ancestors in the time of the Incas. It is remarkable that they should never have discovered the febrifugal qualities of the chinchona.—*London Lancet.*

---

A CURE FOR FISTULA LACHRYMALIS.—M. Delore states, in the "Transections of the Societe des Sciences Medicales of Lyons," that in four cases out of nine, he succeeded in curing his patients by perforating the lachrymal bone, and placing in the cavity thus made a cone composed of arsenical paste. It is, however, difficult to say whether the perforation or the caustic ought to have the merit of the cure.—*London Lancet.*

